# 3-Acetyl-1-(2,3-dichloro­phen­yl)thio­urea

**DOI:** 10.1107/S1600536812030176

**Published:** 2012-07-07

**Authors:** B. Thimme Gowda, Sabine Foro, Sharatha Kumar

**Affiliations:** a Department of Chemistry, Mangalore University, Mangalagangotri 574 199, Mangalore, India; b Institute of Materials Science, Darmstadt University of Technology, Petersenstrasse 23, D-64287 Darmstadt, Germany

## Abstract

In the crystal structure of the title compound, C_9_H_8_Cl_2_N_2_OS, there are two mol­ecules in the asymmetric unit which are connected by a pair of N—H⋯S hydrogen bonds. An intra­molecular N—H⋯O hydrogen bond stabilizes the mol­ecular conformation of each molecule.

## Related literature

For studies on the effects of substituents on the structures and other aspects of *N*-(ar­yl)-amides, see: Gowda *et al.* (2001 ▶); Kumar *et al.* (2012 ▶); Shahwar *et al.* (2012 ▶). For *N*-(ar­yl)-methane­sulfonamides, see: Gowda *et al.* (2007 ▶). For *N*-chloro­aryl­sulfonamides, see: Gowda & Ramachandra (1989 ▶), Shetty & Gowda (2004 ▶).
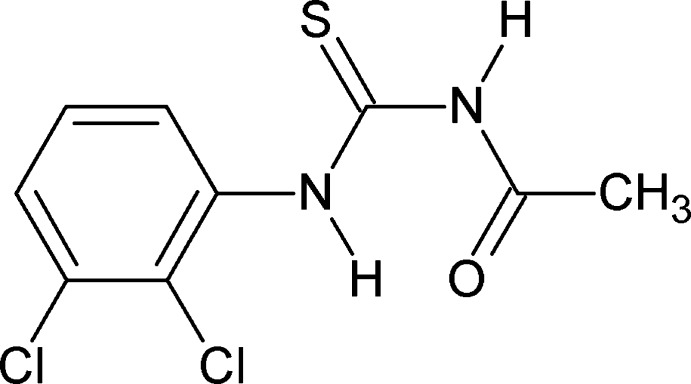


## Experimental

### 

#### Crystal data

C_9_H_8_Cl_2_N_2_OS
*M* *_r_* = 263.13
Triclinic, 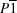

*a* = 7.8475 (6) Å
*b* = 9.5987 (7) Å
*c* = 15.141 (1) Å
α = 90.044 (6)°
β = 91.099 (6)°
γ = 100.208 (6)°
*V* = 1122.24 (14) Å^3^
*Z* = 4
Mo *K*α radiation
μ = 0.74 mm^−1^
*T* = 293 K
0.46 × 0.44 × 0.36 mm

#### Data collection

Oxford Diffraction Xcalibur diffractometer with a Sapphire CCD detector
Absorption correction: multi-scan (*CrysAlis RED*; Oxford Diffraction, 2009 ▶) *T*_min_ = 0.728, *T*_max_ = 0.777
7971 measured reflections
4578 independent reflections
3885 reflections with *I* > 2σ(*I*)
*R*_int_ = 0.011

#### Refinement

*R*[*F* ^2^ > 2σ(*F* ^2^)] = 0.041
*wR*(*F* ^2^) = 0.106
*S* = 1.04
4578 reflections
285 parameters
4 restraints
H atoms treated by a mixture of independent and constrained refinement
Δρ_max_ = 0.67 e Å^−3^
Δρ_min_ = −0.72 e Å^−3^

### 

Data collection: *CrysAlis CCD* (Oxford Diffraction, 2009 ▶); cell refinement: *CrysAlis RED* (Oxford Diffraction, 2009 ▶); data reduction: *CrysAlis RED*; program(s) used to solve structure: *SHELXS97* (Sheldrick, 2008 ▶); program(s) used to refine structure: *SHELXL97* (Sheldrick, 2008 ▶); molecular graphics: *PLATON* (Spek, 2009 ▶); software used to prepare material for publication: *SHELXL97*.

## Supplementary Material

Crystal structure: contains datablock(s) I, global. DOI: 10.1107/S1600536812030176/bt5964sup1.cif

Structure factors: contains datablock(s) I. DOI: 10.1107/S1600536812030176/bt5964Isup2.hkl

Supplementary material file. DOI: 10.1107/S1600536812030176/bt5964Isup3.cml

Additional supplementary materials:  crystallographic information; 3D view; checkCIF report

## Figures and Tables

**Table 1:** Hydrogen-bond geometry (Å, °)

*D*—H⋯*A*	*D*—H	H⋯*A*	*D*⋯*A*	*D*—H⋯*A*
N1—H1N⋯O1	0.85 (2)	1.91 (2)	2.625 (3)	141 (3)
N2—H2N⋯S2	0.84 (2)	2.56 (2)	3.393 (2)	171 (2)
N3—H3N⋯O2	0.81 (2)	1.93 (2)	2.619 (3)	143 (3)
N4—H4N⋯S1	0.84 (2)	2.59 (2)	3.418 (2)	170 (2)

**Fig. 1.**
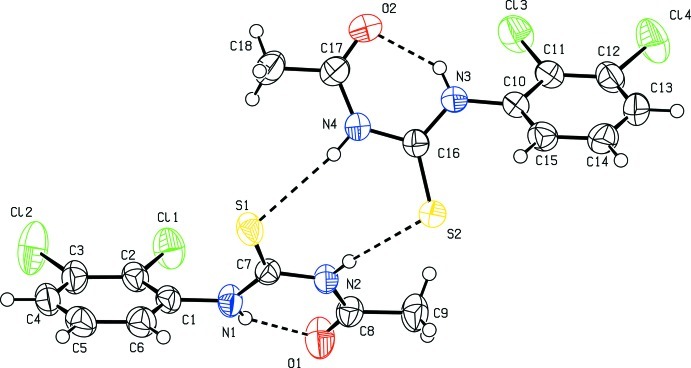
Molecular structure of the title compound, showing the atom labelling scheme and with displacement ellipsoids drawn at the 50% probability level.

**Fig. 2.**
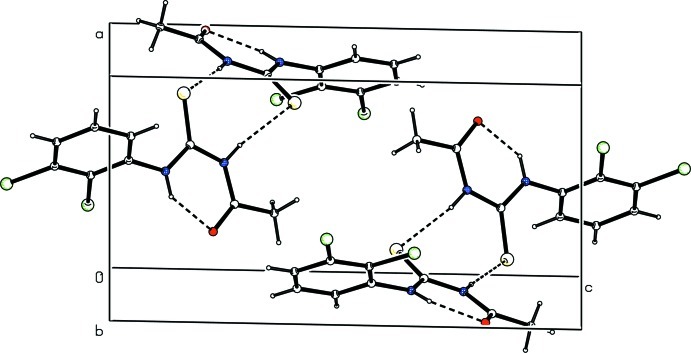
Molecular packing of the title compound with hydrogen bonding shown as dashed lines.

## supplementary crystallographic information

### Comment

As part of studies on the substituent effects on the structures and other aspects of *N*-(aryl)-amides (Gowda *et al.*, 2001; Kumar *et al.*, 2012: Shahwar *et al.*, 2012); *N*-(aryl)-methanesulfonamides (Gowda *et al.*, 2007) and *N*-chloroarylsulfonamides (Gowda & Ramachandra, 1989; Shetty & Gowda, 2004), in the present work, the crystal structure of 3-acetyl-1-(2,3-dichlorophenyl)thiourea has been determined (Fig. 1).

The asymmetric unit of the structure contains two molecules. The conformation of the two N—H bonds are *anti* to each other. Furthermore, the conformations of the amide C═S and the C═O are also *anti* to each other and both the bonds are *anti* to the adjacent N—H bonds, similar to the *anti* conformation observed in 3-acetyl-1-(2,3-dimethylphenyl)thiourea (I)(Kumar *et al.*, 2012). The N—H bond adjacent to the 2,3-dichlorophenyl ring is *syn* to the *ortho*- and *meta*-Cl atoms in one of the molecules and *anti* in the other molecule, compared to the *anti* conformation observed with respect to the *ortho*- and *meta*-methyl groups in the 2,3-dimethylphenyl ring of (I).

The side chains are oriented themselves with respect to the 2,3-dichlorophenyl rings with the torsion angles, C2—C1—N1—C7 = 116.47 (26)° and C6—C1—N1—C7 = -65.77 (33)° in molecule 1 and C11—C10—N3—C16 = 129.96 (25)° and C15—C10—N3—C16 = -53.71 (35)° in molecule 2 of the title compound, compared to the torsion angles of C2—C1—N1—C7 = 83.59 (47)° and C6—C1—N1—C7 = -99.89 (44)° for in (I). The dihedral angles between the phenyl rings and the side chains are 62.5 (1)° and 51.3 (1)°, in the two molecules, compared to the value of 81.33 (10)° in (I).

The hydrogen atoms of the NH attached to the phenyl rings and the amide O atoms are involved in the intramolecular hydrogen bonding. In the crystal, the molecules form inversion dimers through pairs of N—H···S intermolecular hydrogen bonds (Table 1, Fig.2).

### Experimental

3-Acetyl-1-(2,3-dichlorophenyl)thiourea was synthesized by adding a solution of acetyl chloride (0.10 mol) in acetone (30 ml) dropwise to a suspension of ammonium thiocyanate (0.10 mol) in acetone (30 ml). The reaction mixture was refluxed for 30 min. After cooling to room temperature, a solution of 2,3-dichloroaniline (0.10 mol) in acetone (10 ml) was added and refluxed for 3 h. The reaction mixture was poured into acidified cold water. The precipitated title compound was recrystallized to constant melting point from acetonitrile. The purity of the compound was checked and characterized by its infrared spectrum.

Prism like light yellow single crystals used in X-ray diffraction studies were grown in acetonitrile solution by slow evaporation of the solvent at room temperature.

### Refinement

The coordinates of the amino H atoms were refined with the N—H distances restrained to 0.86 (2) Å. H atoms bonded to C were positioned with idealized geometry using a riding model with the aromatic C—H = 0.93 Å, methyl C—H = 0.96 Å. All H atoms were refined with their isotropic displacement parameter set to 1.2 times of the *U*_eq_ of the parent atom.

### Crystal data

**Table:**

C_9_H_8_Cl_2_N_2_OS	*Z* = 4
*M**_r_* = 263.13	*F*(000) = 536
Triclinic, *P*1	*D*_x_ = 1.557 Mg m^−^^3^
Hall symbol: -P 1	Mo *K*α radiation, λ = 0.71073 Å
*a* = 7.8475 (6) Å	Cell parameters from 4895 reflections
*b* = 9.5987 (7) Å	θ = 2.5–27.7°
*c* = 15.141 (1) Å	µ = 0.74 mm^−^^1^
α = 90.044 (6)°	*T* = 293 K
β = 91.099 (6)°	Prism, light yellow
γ = 100.208 (6)°	0.46 × 0.44 × 0.36 mm
*V* = 1122.24 (14) Å^3^	

### Data collection

**Table:**

Oxford Diffraction Xcalibur diffractometer with a Sapphire CCD detector	4578 independent reflections
Radiation source: fine-focus sealed tube	3885 reflections with *I* > 2σ(*I*)
Graphite monochromator	*R*_int_ = 0.011
Rotation method data acquisition using ω scans	θ_max_ = 26.4°, θ_min_ = 2.5°
Absorption correction: multi-scan (*CrysAlis RED*; Oxford Diffraction, 2009)	*h* = −9→8
*T*_min_ = 0.728, *T*_max_ = 0.777	*k* = −11→10
7971 measured reflections	*l* = −18→17

### Refinement

**Table:**

Refinement on *F*^2^	Primary atom site location: structure-invariant direct methods
Least-squares matrix: full	Secondary atom site location: difference Fourier map
*R*[*F*^2^ > 2σ(*F*^2^)] = 0.041	Hydrogen site location: inferred from neighbouring sites
*wR*(*F*^2^) = 0.106	H atoms treated by a mixture of independent and constrained refinement
*S* = 1.04	*w* = 1/[σ^2^(*F*_o_^2^) + (0.0403*P*)^2^ + 0.8845*P*] where *P* = (*F*_o_^2^ + 2*F*_c_^2^)/3
4578 reflections	(Δ/σ)_max_ = 0.002
285 parameters	Δρ_max_ = 0.67 e Å^−^^3^
4 restraints	Δρ_min_ = −0.72 e Å^−^^3^

### Special details

**Table:**

Experimental. CrysAlis RED (Oxford Diffraction, 2009) Empirical absorption correction using spherical harmonics, implemented in SCALE3 ABSPACK scaling algorithm.
Geometry. All e.s.d.'s (except the e.s.d. in the dihedral angle between two l.s. planes) are estimated using the full covariance matrix. The cell e.s.d.'s are taken into account individually in the estimation of e.s.d.'s in distances, angles and torsion angles; correlations between e.s.d.'s in cell parameters are only used when they are defined by crystal symmetry. An approximate (isotropic) treatment of cell e.s.d.'s is used for estimating e.s.d.'s involving l.s. planes.
Refinement. Refinement of *F*^2^ against ALL reflections. The weighted *R*-factor *wR* and goodness of fit *S* are based on *F*^2^, conventional *R*-factors *R* are based on *F*, with *F* set to zero for negative *F*^2^. The threshold expression of *F*^2^ > σ(*F*^2^) is used only for calculating *R*-factors(gt) *etc*. and is not relevant to the choice of reflections for refinement. *R*-factors based on *F*^2^ are statistically about twice as large as those based on *F*, and *R*- factors based on ALL data will be even larger.

### Fractional atomic coordinates and isotropic or equivalent isotropic displacement parameters (Å^2^)

**Table:**

	*x*	*y*	*z*	*U*_iso_*/*U*_eq_	
Cl1	0.39378 (10)	0.64301 (8)	−0.04620 (5)	0.0675 (2)	
Cl2	0.51017 (13)	0.81095 (13)	−0.21801 (5)	0.0956 (3)	
S1	0.86892 (8)	0.69266 (7)	0.15722 (5)	0.05361 (18)	
O1	0.3014 (2)	0.6735 (2)	0.21951 (13)	0.0687 (6)	
N1	0.5647 (3)	0.7665 (2)	0.11819 (13)	0.0457 (5)	
H1N	0.458 (2)	0.754 (3)	0.1305 (18)	0.055*	
N2	0.5747 (2)	0.6306 (2)	0.24233 (12)	0.0397 (4)	
H2N	0.639 (3)	0.591 (3)	0.2760 (15)	0.048*	
C1	0.6232 (3)	0.8420 (2)	0.04055 (15)	0.0412 (5)	
C2	0.5487 (3)	0.7949 (3)	−0.04025 (16)	0.0441 (5)	
C3	0.5999 (3)	0.8705 (3)	−0.11636 (17)	0.0526 (6)	
C4	0.7246 (3)	0.9910 (3)	−0.11191 (19)	0.0561 (7)	
H4	0.7589	1.0412	−0.1630	0.067*	
C5	0.7983 (3)	1.0369 (3)	−0.0314 (2)	0.0564 (7)	
H5	0.8827	1.1182	−0.0283	0.068*	
C6	0.7479 (3)	0.9632 (3)	0.04515 (18)	0.0512 (6)	
H6	0.7977	0.9951	0.0993	0.061*	
C7	0.6603 (3)	0.6990 (2)	0.17092 (14)	0.0374 (5)	
C8	0.4039 (3)	0.6220 (3)	0.26475 (16)	0.0451 (5)	
C9	0.3545 (3)	0.5463 (3)	0.34875 (18)	0.0607 (7)	
H9A	0.2336	0.5439	0.3587	0.073*	
H9B	0.4214	0.5949	0.3969	0.073*	
H9C	0.3768	0.4513	0.3449	0.073*	
Cl3	0.68592 (11)	−0.06753 (7)	0.35412 (5)	0.0693 (2)	
Cl4	0.60530 (13)	−0.20874 (9)	0.53971 (7)	0.0884 (3)	
S2	0.78757 (8)	0.45828 (6)	0.39216 (4)	0.04733 (16)	
O2	1.0172 (3)	0.1780 (2)	0.20337 (14)	0.0772 (6)	
N3	0.9002 (3)	0.2163 (2)	0.36075 (13)	0.0453 (5)	
H3N	0.932 (3)	0.169 (3)	0.3223 (15)	0.054*	
N4	0.9362 (3)	0.3818 (2)	0.24969 (13)	0.0432 (4)	
H4N	0.924 (3)	0.463 (2)	0.2336 (17)	0.052*	
C10	0.8602 (3)	0.1564 (2)	0.44498 (15)	0.0405 (5)	
C11	0.7626 (3)	0.0208 (2)	0.44964 (16)	0.0440 (5)	
C12	0.7286 (3)	−0.0417 (3)	0.53190 (18)	0.0531 (6)	
C13	0.7920 (4)	0.0296 (3)	0.60792 (18)	0.0591 (7)	
H13	0.7690	−0.0126	0.6627	0.071*	
C14	0.8890 (4)	0.1627 (3)	0.60259 (17)	0.0570 (7)	
H14	0.9317	0.2107	0.6540	0.068*	
C15	0.9241 (3)	0.2264 (3)	0.52160 (16)	0.0494 (6)	
H15	0.9908	0.3166	0.5187	0.059*	
C16	0.8777 (3)	0.3440 (2)	0.33372 (14)	0.0378 (5)	
C17	1.0071 (3)	0.3013 (3)	0.18971 (17)	0.0509 (6)	
C18	1.0707 (4)	0.3771 (3)	0.10721 (18)	0.0633 (7)	
H18A	1.0801	0.3093	0.0618	0.076*	
H18B	1.1822	0.4344	0.1187	0.076*	
H18C	0.9906	0.4364	0.0882	0.076*	

### Atomic displacement parameters (Å^2^)

**Table:**

	*U*^11^	*U*^22^	*U*^33^	*U*^12^	*U*^13^	*U*^23^
Cl1	0.0655 (4)	0.0654 (4)	0.0644 (4)	−0.0075 (3)	−0.0080 (3)	0.0111 (3)
Cl2	0.0922 (6)	0.1402 (9)	0.0462 (4)	−0.0007 (6)	−0.0098 (4)	0.0234 (5)
S1	0.0414 (3)	0.0606 (4)	0.0619 (4)	0.0158 (3)	0.0148 (3)	0.0243 (3)
O1	0.0413 (10)	0.1009 (16)	0.0665 (12)	0.0190 (10)	0.0075 (9)	0.0325 (11)
N1	0.0369 (10)	0.0585 (12)	0.0425 (11)	0.0101 (9)	0.0053 (8)	0.0153 (9)
N2	0.0370 (10)	0.0457 (10)	0.0369 (10)	0.0085 (8)	0.0031 (8)	0.0084 (8)
C1	0.0373 (11)	0.0450 (12)	0.0440 (12)	0.0140 (9)	0.0063 (9)	0.0114 (10)
C2	0.0390 (12)	0.0481 (13)	0.0469 (13)	0.0124 (10)	0.0013 (10)	0.0109 (10)
C3	0.0494 (14)	0.0666 (16)	0.0448 (13)	0.0184 (12)	0.0053 (11)	0.0165 (12)
C4	0.0547 (15)	0.0618 (16)	0.0570 (16)	0.0228 (13)	0.0183 (12)	0.0259 (13)
C5	0.0490 (14)	0.0439 (13)	0.0766 (19)	0.0075 (11)	0.0175 (13)	0.0136 (12)
C6	0.0496 (14)	0.0492 (14)	0.0549 (15)	0.0084 (11)	0.0046 (11)	0.0040 (11)
C7	0.0406 (11)	0.0359 (11)	0.0352 (11)	0.0054 (9)	0.0019 (9)	−0.0002 (8)
C8	0.0404 (12)	0.0504 (13)	0.0435 (13)	0.0054 (10)	0.0047 (10)	0.0039 (10)
C9	0.0482 (14)	0.0785 (19)	0.0559 (16)	0.0111 (13)	0.0135 (12)	0.0228 (14)
Cl3	0.0901 (5)	0.0490 (4)	0.0630 (4)	−0.0018 (3)	−0.0183 (4)	−0.0066 (3)
Cl4	0.1007 (6)	0.0566 (4)	0.0989 (7)	−0.0115 (4)	0.0078 (5)	0.0281 (4)
S2	0.0601 (4)	0.0442 (3)	0.0409 (3)	0.0175 (3)	0.0060 (3)	0.0027 (2)
O2	0.1154 (18)	0.0608 (13)	0.0642 (13)	0.0367 (12)	0.0298 (12)	0.0009 (10)
N3	0.0624 (13)	0.0365 (10)	0.0384 (10)	0.0122 (9)	0.0063 (9)	−0.0002 (8)
N4	0.0492 (11)	0.0402 (10)	0.0407 (10)	0.0088 (9)	0.0072 (8)	0.0048 (8)
C10	0.0456 (12)	0.0361 (11)	0.0413 (12)	0.0115 (9)	0.0024 (9)	0.0030 (9)
C11	0.0471 (13)	0.0383 (12)	0.0470 (13)	0.0096 (10)	−0.0026 (10)	0.0009 (10)
C12	0.0540 (14)	0.0435 (13)	0.0625 (16)	0.0096 (11)	0.0084 (12)	0.0135 (11)
C13	0.0730 (18)	0.0640 (17)	0.0450 (14)	0.0233 (14)	0.0111 (13)	0.0147 (12)
C14	0.0720 (18)	0.0622 (16)	0.0406 (13)	0.0232 (14)	−0.0027 (12)	−0.0037 (11)
C15	0.0575 (15)	0.0424 (13)	0.0480 (14)	0.0082 (11)	−0.0027 (11)	−0.0019 (10)
C16	0.0360 (11)	0.0369 (11)	0.0385 (11)	0.0015 (9)	−0.0010 (9)	0.0004 (9)
C17	0.0534 (14)	0.0553 (15)	0.0454 (13)	0.0127 (12)	0.0066 (11)	−0.0024 (11)
C18	0.0667 (17)	0.078 (2)	0.0479 (15)	0.0187 (15)	0.0160 (13)	0.0028 (13)

### Geometric parameters (Å, º)

**Table:**

Cl1—C2	1.726 (2)	Cl3—C11	1.719 (2)
Cl2—C3	1.734 (3)	Cl4—C12	1.725 (3)
S1—C7	1.666 (2)	S2—C16	1.669 (2)
O1—C8	1.217 (3)	O2—C17	1.218 (3)
N1—C7	1.330 (3)	N3—C16	1.332 (3)
N1—C1	1.422 (3)	N3—C10	1.417 (3)
N1—H1N	0.846 (17)	N3—H3N	0.808 (17)
N2—C8	1.378 (3)	N4—C17	1.379 (3)
N2—C7	1.387 (3)	N4—C16	1.388 (3)
N2—H2N	0.843 (16)	N4—H4N	0.836 (16)
C1—C6	1.382 (3)	C10—C15	1.381 (3)
C1—C2	1.386 (3)	C10—C11	1.391 (3)
C2—C3	1.390 (3)	C11—C12	1.392 (3)
C3—C4	1.378 (4)	C12—C13	1.377 (4)
C4—C5	1.377 (4)	C13—C14	1.370 (4)
C4—H4	0.9300	C13—H13	0.9300
C5—C6	1.385 (4)	C14—C15	1.381 (4)
C5—H5	0.9300	C14—H14	0.9300
C6—H6	0.9300	C15—H15	0.9300
C8—C9	1.489 (3)	C17—C18	1.495 (4)
C9—H9A	0.9600	C18—H18A	0.9600
C9—H9B	0.9600	C18—H18B	0.9600
C9—H9C	0.9600	C18—H18C	0.9600
			
C7—N1—C1	125.50 (19)	C16—N3—C10	126.43 (19)
C7—N1—H1N	114.5 (19)	C16—N3—H3N	114 (2)
C1—N1—H1N	119.6 (19)	C10—N3—H3N	120 (2)
C8—N2—C7	128.45 (19)	C17—N4—C16	128.0 (2)
C8—N2—H2N	117.7 (18)	C17—N4—H4N	116.8 (19)
C7—N2—H2N	113.8 (18)	C16—N4—H4N	115.1 (19)
C6—C1—C2	120.1 (2)	C15—C10—C11	119.8 (2)
C6—C1—N1	121.0 (2)	C15—C10—N3	121.4 (2)
C2—C1—N1	118.9 (2)	C11—C10—N3	118.8 (2)
C1—C2—C3	119.6 (2)	C10—C11—C12	119.3 (2)
C1—C2—Cl1	120.12 (18)	C10—C11—Cl3	119.74 (18)
C3—C2—Cl1	120.3 (2)	C12—C11—Cl3	120.92 (19)
C4—C3—C2	120.4 (2)	C13—C12—C11	120.4 (2)
C4—C3—Cl2	119.6 (2)	C13—C12—Cl4	119.3 (2)
C2—C3—Cl2	120.0 (2)	C11—C12—Cl4	120.3 (2)
C5—C4—C3	119.6 (2)	C14—C13—C12	119.8 (2)
C5—C4—H4	120.2	C14—C13—H13	120.1
C3—C4—H4	120.2	C12—C13—H13	120.1
C4—C5—C6	120.6 (2)	C13—C14—C15	120.7 (2)
C4—C5—H5	119.7	C13—C14—H14	119.7
C6—C5—H5	119.7	C15—C14—H14	119.7
C1—C6—C5	119.7 (3)	C10—C15—C14	120.0 (2)
C1—C6—H6	120.2	C10—C15—H15	120.0
C5—C6—H6	120.2	C14—C15—H15	120.0
N1—C7—N2	115.39 (19)	N3—C16—N4	115.5 (2)
N1—C7—S1	125.13 (17)	N3—C16—S2	125.53 (17)
N2—C7—S1	119.48 (16)	N4—C16—S2	118.93 (16)
O1—C8—N2	122.4 (2)	O2—C17—N4	122.3 (2)
O1—C8—C9	122.6 (2)	O2—C17—C18	122.8 (2)
N2—C8—C9	115.0 (2)	N4—C17—C18	114.9 (2)
C8—C9—H9A	109.5	C17—C18—H18A	109.5
C8—C9—H9B	109.5	C17—C18—H18B	109.5
H9A—C9—H9B	109.5	H18A—C18—H18B	109.5
C8—C9—H9C	109.5	C17—C18—H18C	109.5
H9A—C9—H9C	109.5	H18A—C18—H18C	109.5
H9B—C9—H9C	109.5	H18B—C18—H18C	109.5
			
C7—N1—C1—C6	−65.8 (3)	C16—N3—C10—C15	−53.7 (3)
C7—N1—C1—C2	116.5 (3)	C16—N3—C10—C11	130.0 (2)
C6—C1—C2—C3	−0.1 (3)	C15—C10—C11—C12	0.9 (3)
N1—C1—C2—C3	177.6 (2)	N3—C10—C11—C12	177.3 (2)
C6—C1—C2—Cl1	179.65 (18)	C15—C10—C11—Cl3	−178.97 (19)
N1—C1—C2—Cl1	−2.6 (3)	N3—C10—C11—Cl3	−2.6 (3)
C1—C2—C3—C4	0.4 (4)	C10—C11—C12—C13	−0.4 (4)
Cl1—C2—C3—C4	−179.44 (19)	Cl3—C11—C12—C13	179.4 (2)
C1—C2—C3—Cl2	178.94 (18)	C10—C11—C12—Cl4	179.07 (18)
Cl1—C2—C3—Cl2	−0.9 (3)	Cl3—C11—C12—Cl4	−1.1 (3)
C2—C3—C4—C5	−0.2 (4)	C11—C12—C13—C14	0.0 (4)
Cl2—C3—C4—C5	−178.8 (2)	Cl4—C12—C13—C14	−179.5 (2)
C3—C4—C5—C6	−0.2 (4)	C12—C13—C14—C15	0.0 (4)
C2—C1—C6—C5	−0.2 (4)	C11—C10—C15—C14	−0.9 (4)
N1—C1—C6—C5	−177.9 (2)	N3—C10—C15—C14	−177.2 (2)
C4—C5—C6—C1	0.4 (4)	C13—C14—C15—C10	0.5 (4)
C1—N1—C7—N2	−179.0 (2)	C10—N3—C16—N4	176.4 (2)
C1—N1—C7—S1	1.4 (4)	C10—N3—C16—S2	−3.3 (4)
C8—N2—C7—N1	1.1 (3)	C17—N4—C16—N3	3.3 (3)
C8—N2—C7—S1	−179.31 (19)	C17—N4—C16—S2	−177.0 (2)
C7—N2—C8—O1	2.5 (4)	C16—N4—C17—O2	4.5 (4)
C7—N2—C8—C9	−177.0 (2)	C16—N4—C17—C18	−175.3 (2)

### Hydrogen-bond geometry (Å, º)

**Table:**

*D*—H···*A*	*D*—H	H···*A*	*D*···*A*	*D*—H···*A*
N1—H1*N*···O1	0.85 (2)	1.91 (2)	2.625 (3)	141 (3)
N2—H2*N*···S2	0.84 (2)	2.56 (2)	3.393 (2)	171 (2)
N3—H3*N*···O2	0.81 (2)	1.93 (2)	2.619 (3)	143 (3)
N4—H4*N*···S1	0.84 (2)	2.59 (2)	3.418 (2)	170 (2)
